# Serum and Plasma Metabolomic Biomarkers for Lung Cancer

**DOI:** 10.6026/97320630013202

**Published:** 2017-06-30

**Authors:** Nishith Kumar, Md. Shahjaman, Md. Nurul Haque Mollah, S. M. Shahinul Islam, Md. Aminul Hoque

**Affiliations:** 1 Bioinformatics Lab, Department of Statistics, Rajshahi University, Rajshahi, Bangladesh; 2 Department of Statistics, Bangabandhu Sheikh Mujibur Rahman Science and Technology University, Gopalganj, Bangladesh; 3 Department of Statistics, Begum Rokeya University, Rangpur, Bangladesh; 4 Institute of Biological Sciences, Rajshahi University, Rajshahi, Bangladesh

**Keywords:** Metabolomics, biomarker identification, Student t-test, Kruskal-Wallis test, support vector machine (SVM), pathway analysis

## Abstract

In drug invention and early disease prediction of lung cancer, metabolomic biomarker detection is very important. Mortality rate can be decreased, if cancer is predicted at the earlier stage. Recent diagnostic techniques for lung cancer are not prognosis diagnostic techniques. However, if we know the name of the metabolites, whose intensity levels are considerably changing between cancer subject and control subject, then it will be easy to early diagnosis the disease as well as to discover the drug. Therefore, in this paper we have identified the influential plasma and serum blood sample metabolites for lung cancer and also identified the biomarkers that will be helpful for early disease prediction as well as for drug invention. To identify the influential metabolites, we considered a parametric and a nonparametric test namely student׳s t-test as parametric and Kruskal-Wallis test as non-parametric test. We also categorized the up-regulated and down-regulated metabolites by the heatmap plot and identified the biomarkers by support vector machine (SVM) classifier and pathway analysis. From our analysis, we got 27 influential (p-value<0.05) metabolites from plasma sample and 13 influential (p-value<0.05) metabolites from serum sample. According to the importance plot through SVM classifier, pathway analysis and correlation network analysis, we declared 4 metabolites (taurine, aspertic acid, glutamine and pyruvic acid) as plasma biomarker and 3 metabolites (aspartic acid, taurine and inosine) as serum biomarker.

## Background

Lung cancer is the leading cause of cancer mortality in United States as well as all over the world [1]. In 2012, 1.8 million people were affected by lung cancer and 1.6 million deaths [2] worldwide. This is the most common cause of cancer-related death in men and second most common in women after breast cancer [3]. Early diagnosis of lung cancer can increase the survival rate at 85% [4]. Till now, there are no FDA-approved diagnostic tests available for detecting the existence of lung cancer [5]. However, Due to the development of molecular biology early diagnosis of cancer is possible through metabolomics data analysis.

Metabolomics is the powerful high throughput technology based on the entire set of metabolites that provide potential information because it measures and quantify the end product of cellular metabolism. Any disorder of cellular process is revealed with the changes of metabolites level. Therefore, differentially expressed (DE) metabolites identification between normal and cancer patient is very important for early diagnosis of disease as well as metabolomic biomarker discovery. There are several parametric and non-parametric approaches for DE metabolite identification. The well-known parametric tests are student׳s t-test, F-test, significant analysis of microarray (SAM), EBarrays, BRIDGE, LIMMA etc. and non-parametric tests are Wilcoxon signed-rank test, Mann-Whitney test, Kruskal-Wallis test etc. However, biologist often use fold change method for influential metabolite identification. We generally use parametric test if the data follow the normality property, on the contrary, if the dataset contain outliers and non-normality properties then non-parametric test is applicable. Student׳s t-test has been used for different cancer biomarker identification namely - prostate cancer [6], colon carcinoma [7], pancreatic cancer [8], kidney cancer [9], hepatocellular carcinoma [10] etc. Metabolomics dataset often contain outliers due to several steps involves in the data generating processes [11]. Therefore, to identify the DE metabolite for lung cancer, we used both t-test as parametric test and Kruskal-Wallis test as non-parametric test for identifying DE metabolite for lung cancer. We declare those metabolites as DE, which are significant in any of the methods.

In this paper, we also classified the up regulated and downregulated metabolites by using cluster heatmap plot [12]. Upregulated and down-regulated metabolites for lung cancer are important to early diagnosis the disease, for drug discovery and biomarker discovery. On the basis of heatmap plot, importance plot (importance score is calculated using SVM classifier with radial basis kernel function), pathway analysis and correlation network plot finally we identified the biomarker metabolites.

In this paper, we took plasma and serum blood samples for identifying the significant metabolites and biomarker discovery. Here, we got 27 significant (p-value<0.05) metabolites from plasma sample and 13 significant (p-value<0.05) metabolites from serum sample for lung cancer. According to the importance plot, pathway analysis and metabolomic correlation network analysis, we declared 4 metabolites (taurine, aspertic acid, glutamine and pyruvic acid) as plasma biomarker and 3 metabolites (aspartic acid, taurine and inosine) as serum biomarker for lung cancer. Among these metabolites taurine, aspartic acid and pyruvic acid are up regulated and glutamine and inosine are down regulated in cancer patient.

## Methodology

In this paper, we have identified the significant metabolites from plasma and serum samples using student׳s t-test and Kruskal- Wallis test. This test has been implemented by R-software using function t.test and kruskal.test. Heatmap plot and importance plot using SVM have also been implemented by R-software in library gplots and caret respectively. We did pathway analysis by online software MetaboAnalyst 3.0 [13]. The detail description of the analyzed dataset and the significant metabolite identification methods namely student׳s t-test and Kruskal-Wallis test are below.

### Student׳s t-test

Let us consider a random sample x11, x12, ... x1 n1 follows normal distribution with mean μ1 and variance σ12 (i.e., N(μ1, σ12)) and another independent random sample x21, x22, ... x2 n2 follows N(μ2, σ22). If we want to test the hypothesis H0: μ1= μ2 Vs H1: μ1 ≠ μ2

The test statistic (for σ12= σ22) is,

$t=\frac{{\mathrm{x̄}}_{1}{\mathrm{- x̄}}_{2}}{\surd s^{2}(\frac{1}{n_{1}}+\frac{1}{n_{2}})}$

Here,

${\mathrm{x̄}}_{1}=\Sigma \frac{x_{1i}}{n_{1}};{\mathrm{x̄}}_{2}=\Sigma \frac{x_{2i}}{n_{2}};{s_{1}}^{2}=\frac{1}{n_{1}-1}\Sigma (x_{1i}{\mathrm{- x̄}}_{1}{)}^{2};{s_{2}}^{2}=\frac{1}{n_{2}-1}\Sigma (x_{2i}{\mathrm{- x̄}}_{2}{)}^{2};$

and

$s^{2}=\frac{(n_{1}\mathrm{- 1}){s_{1}}^{2}+(n_{2}\mathrm{- 1}){s_{2}}^{2}}{n_{1}+n_{2}\mathrm{- 2}}$

The calculated t value is compared with the tabulated t-value with n1 + n2 - 2 degrees of freedom.

If σ12 ≠ σ22; the test statistic is,

$t=\frac{{\mathrm{x̄}}_{1}{\mathrm{- x̄}}_{2}}{\surd (\frac{{s_{1}}^{2}}{n_{1}}+\frac{{s_{2}}^{2}}{n_{2}})}$

In both cases, the Ho will be rejected at α % level of significance, if the calculated t value is greater than the tabulated t value with n1 + n2 - 2 degrees of freedom and α % level of significance.

If X is a metabolomics data matrix that contains two types of samples (e.g., cancer vs. control), then for the ith metabolite xij; j=1,2, . . . n1 is a sample for type-1 (e.g., cancer) with sample size n1 and xik; k=1,2, . . ., n2 , is the sample for type-2(e.g., control) with sample size n2 ; we assume Ho, “the ith metabolite is not differentially expressed between cancer vs. control group”. Usually Ho is rejected if ׳p-value׳ <0.05.

### Kruskal-Wallis

This is a non-parametric test was proposed by Kruskal and Wallis [14] and it is used when the data does not satisfy the normality property and contains outliers. The test statistic of Kruskal- Wallis for k groups each of size ni is defined by

$T=\frac{1}{s^{2}}[\overset{k}{\underset{\mathrm{i=1}}{\Sigma }}\frac{R_{i}}{n_{i}}\mathrm{-N}\frac{{\mathrm{(N+1)}}^{2}}{4}]$

where, N is the total number and Ri is the sum of the ranks for the i-th sample and

$s^{2}=\frac{1}{N-1}[\overset{}{\underset{\mathrm{i,j}}{\Sigma }}{R_{\mathrm{ij}}}^{2}\mathrm{-N}\frac{{\mathrm{(N+1)}}^{2}}{4}]$

we assume Ho is that all k distribution functions are equal.

### Dataset Description

The dataset used in this paper was produced by Gas chromatography time of flight mass spectrometry (GC-TOF-MS) using the blood sample of 82 subjects (20 males and 62 females). All samples were collected Among the 82 subjects, 41 blood samples came from the patients with lung cancer and another 41 samples were taken from the individuals without cancer. These blood samples were acquired from the bio-repositories of two institutes (Fred Hutchison Cancer Research Center (FHCRC) and University of California at Davis Medical Center (UCDMC)). All samples were collected with individuals consent and followed the IRB protocols, which was approved by each Institution׳s Institutional Review Board and its aim was to use only for research purposes. Blood samples were collected using EDTA tubes and prepared the samples (serum and plasma) using approved protocols and stored at -80 °C. Raw data of GC-TOFMS were processed using ChromaTOF software (v. 2.32) for peak finding and mass spectral deconvolution. Result files were exported and filtered for consistency using the UC Davis Metabolomics BinBase database. Finally, 158 metabolites were identified as known metabolites. Thus, the dataset contain 82 subjects (41 cancer and 41 control) and 158 metabolites. This dataset had been produced by Oliver Fiehn, whose study ID was ST000392 [5]. We used log2 transformation and auto-scaling to normalize the dataset.

## Results and Discussion

**Figure 1:**
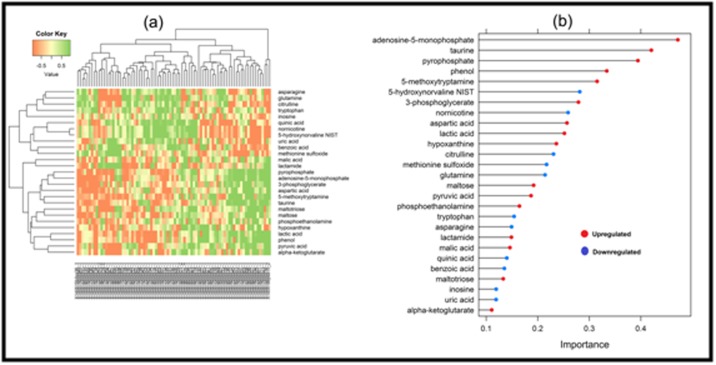
Heatmap plot (a), importance plot (b), using the significant metabolites of plasma sample for lung cancer.

**Figure 2:**
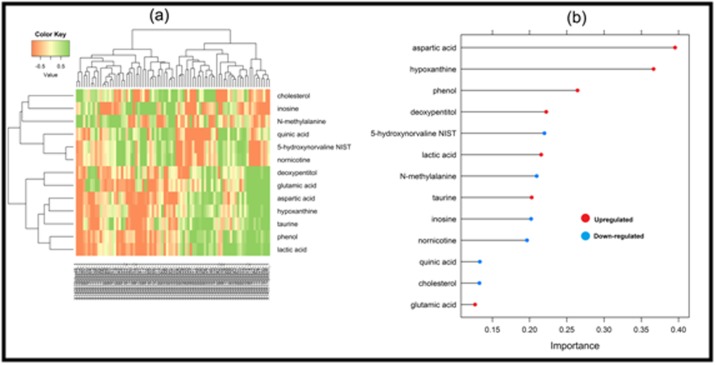
Heatmap plot (a), importance plot (b), using the significant metabolites of serum sample for lung cancer.

**Figure 3:**
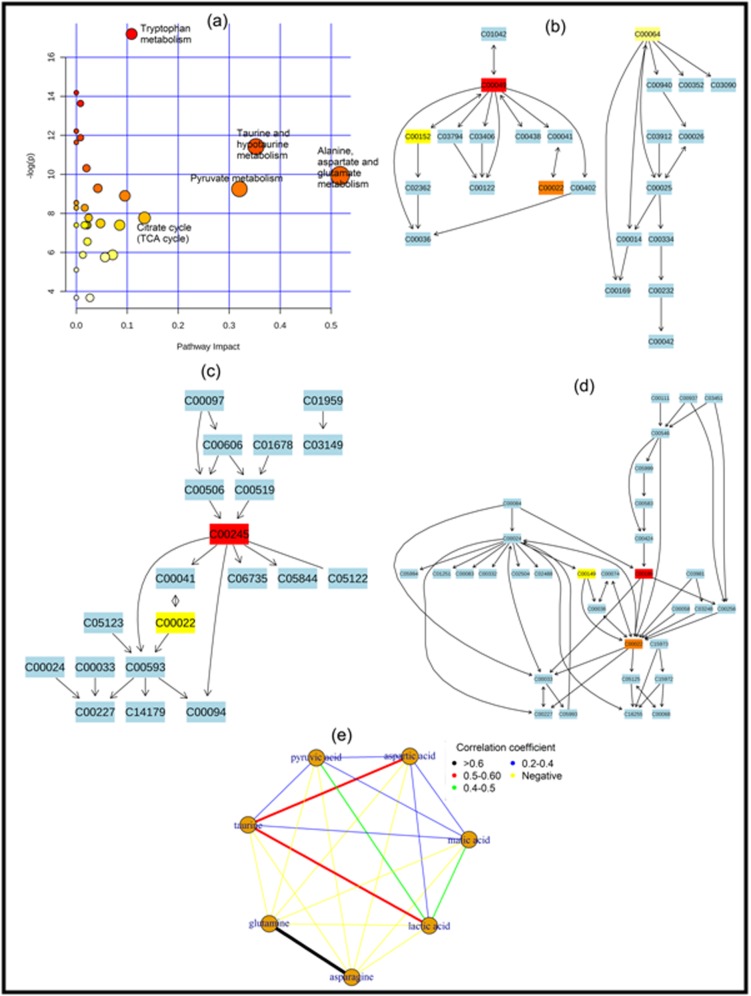
Plasma biomarker identification for lung cancer using pathway analysis plot (a), alanine, aspartate and glutamate metabolism pathway (b), taurine and hypotaurine metabolism pathway (c), pyruvate metabolism pathway (d) and correlation network plot (e).

**Figure 4:**
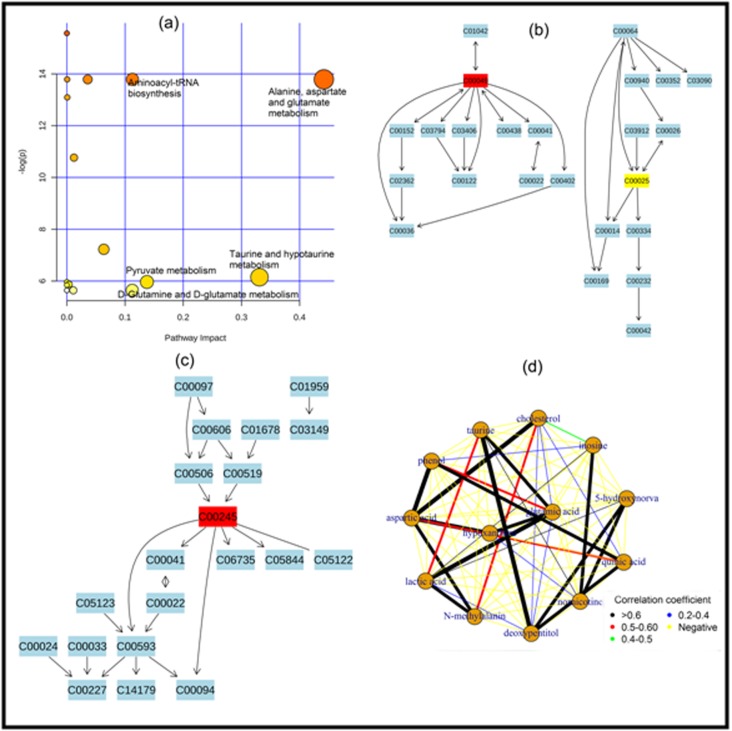
Serum biomarker identification for lung cancer using pathway analysis plot(a), alanine, aspartate and glutamate metabolism pathway(b), taurine and hypotaurine metabolism pathway (c), and correlation network plot (d).

To identify the significant metabolites from the plasma and serum blood samples for lung cancer, we used Student׳s t-test and Kruskal -Wallis test, where, p-values were adjusted using Benjamini-Hochberg (BH) procedure. The lists of significant metabolites of plasma and serum samples for lung cancer are given in Table 1. Table 1 shows that 27 significant metabolites were identified from the plasma samples for lung cancer, among those significant metabolites, t-test identified 24 and Kruskal- Wallis test identified 25 significant metabolites (BH adjusted pvalue< 0.05). On the contrary, 13 significant metabolites were identified from the serum samples for lung cancer, among these 13 significant metabolites, t-test identified 12 and Kruskal-Wallis test identified 11 significant metabolites (BH adjusted pvalue< 0.05). Using the identified significant metabolites, we also drew the heatmap plot to classify the up regulated and downregulated metabolites for plasma and serum sample, we also ranked those metabolites according to the importance (importance score is calculated using SVM classifier with radial basis kernel function), which were depicted in Figure 1 and Figure 2 respectively. Furthermore, we analyzed the pathway and drew the correlation network plot to identify the plasma and serum biomarker for lung cancer. Figure 3 and Figure 4 contain the pathway analysis plot and correlation network plot to identify the plasma and serum biomarker for lung cancer. From Figure 3 (a), we got three important metabolomic pathway for plasma biomarker namely (i) alanine, aspartate and glutamate metabolism pathway, (ii) taurine and hypotaurine metabolism pathway and (iii) pyruvate metabolism pathway. Also from Figure 4 (a), we got two important metabolomic pathway for serum biomarker: (i) alanine, aspartate and glutamate metabolism pathway and (ii) taurine and hypotaurine metabolism pathway. According to the importance plot (Figure 1 and Figure 2), pathway analysis and metabolomic correlation network analysis (Figure 3 and Figure 4), we declared 4 metabolites (taurine, aspertic acid, glutamine and pyruvic acid) as plasma biomarker and 3 metabolites (aspartic acid, taurine and inosine) as serum biomarker for lung cancer. Among these metabolites taurine, aspartic acid and pyruvic acid are up regulated and glutamine and inosine are down regulated in cancer patient. This is the dry laboratory based untargeted metabolomics results. To get the final and more accurate results, further analysis could be the wet laboratory experiment for targeted metabolomics analysis.

## Conclusion

We analysed GC-TOF-MS based untargeted metabolomics data of plasma and serum blood samples. Blood samples were collected from 41 lung cancer cases and 41 control subjects to identify the significant metabolites as well as to discover the plasma and serum biomarker for lung cancer. In our analysis, we got 27 significant metabolites (BH adjusted p-value<0.05) from plasma samples and 13 significant metabolites (BH adjusted pvalue< 0.05) for serum samples for lung cancer. We also got 3 important pathway: (i) alanine, aspartate and glutamate metabolism pathway, (ii) taurine and hypotaurine metabolism pathway and (iii) pyruvate metabolism pathway from plasma samples and 2 important pathway: (i) alanine, aspartate and glutamate metabolism pathway and (ii) taurine and hypotaurine metabolism pathway from serum samples for lung cancer. On the basis of the importance plot, pathway analysis and metabolomic correlation network analysis, we declared 4 metabolites (taurine, aspertic acid, glutamine and pyruvic acid) as plasma biomarker and 3 metabolites (aspartic acid, taurine and inosine) as serum biomarker for lung cancer. Among these metabolites taurine, aspartic acid and pyruvic acid are up regulated and glutamine and inosine are down regulated in cancer patient. We think, this analysis could be helpful for targeted metabolomics researcher, who may validate the result by wet laboratory experiment.

## Figures and Tables

**Table 1:** List of significant metabolites of plasma and serum samples for lung cancer.

Significant Metabolite Name	KEGG ID	Raw p-Value of t-test	BH Adjusted p-Value of t-test	Raw p-Value of Kruskal- Wallis	BH Adjusted p-Value of Kruskal- Wallis
Plasma Sample				
3-phosphoglycerate	C00597	3.34E-06	0.00011	4.31E-06	0.00013
5-hydroxynorvaline NIST	-	0.00017	0.00272	0.00022	0.00346
5-methoxytryptamine	C05659	1.28E-07	6.76E-06	3.77E-06	0.00013
adenosine-5-monophosphate	-	6.93E-12	1.10E-09	1.17E-09	1.85E-07
alpha-ketoglutarate	-	0.00412	0.02956	0.0063	0.04151
asparagine	C00152	0.00093	0.00984	0.00221	0.01837
aspartic acid	C00049	5.18E-06	0.00014	9.29E-06	0.00021
benzoic acid	C00180	0.00145	0.01352	0.00416	0.02987
citrulline	C00327	0.0006	0.00698	0.00036	0.00433
hypoxanthine	C00262	0.00352	0.02647	0.00186	0.01633
lactic acid	C00186	9.39E-06	0.00021	2.30E-05	0.00045
malic acid	C00149	0.00117	0.01155	0.0018	0.01633
maltose	C00208	0.00011	0.00187	0.00017	0.00302
maltotriose	C01835	0.00546	0.03747	0.00952	0.05573
methionine sulfoxide	-	0.00033	0.00429	0.00055	0.00621
nornicotine	C06524	0.00022	0.00321	0.00026	0.00372
phenol	C00146	8.03E-05	0.00158	5.51E-06	0.00014
phosphoethanolamine	C00346	0.00295	0.02328	0.00151	0.01592
pyrophosphate	C00013	1.20E-08	9.48E-07	1.04E-07	8.25E-06
pyruvic acid	C00022	0.00062	0.00698	0.00028	0.00372
quinic acid	C06746	0.00175	0.01538	0.00278	0.02197
taurine	C00245	1.54E-06	6.07E-05	6.99E-07	3.68E-05
tryptophan	C00078	0.00638	0.04201	0.01149	0.06265
uric acid	C00366	0.00264	0.02199	0.00386	0.02909
glutamine	C00064	0.02537	0.12059	0.00164	0.0162
inosine	C00294	0.00835	0.05203	0.00561	0.03853
lactamide	-	0.0109	0.0594	0.00756	0.04779
	Serum Sample				
5-hydroxynorvaline NIST	-	0.00107	0.04239	0.00192	0.04333
aspartic acid	C00049	4.11E-07	6.50E-05	2.10E-06	0.00016
cholesterol	C00187	0.00285	0.04286	0.00286	0.04509
glutamic acid	C00025	0.00366	0.04814	0.00596	0.07248
hypoxanthine	C00262	0.00015	0.01197	1.63E-06	0.00016
inosine	C00294	0.00054	0.02862	0.00039	0.01562
lactic acid	C00186	0.00263	0.04286	0.0018	0.04333
N-methylalanine	-	0.00195	0.04286	0.00314	0.04509
nornicotine	C06524	0.00237	0.04286	0.00416	0.05476
phenol	C00146	0.00298	0.04286	0.00028	0.01488
quinic acid	C06746	0.00145	0.04286	0.00238	0.04454
taurine	C00245	0.00216	0.04286	0.00053	0.01682
deoxypentitol	-	0.01619	0.12789	0.00254	0.04454

## References

[1] http://www.cancer.org/research/cancerfactsstatistics/cancerfactsfigures2014/index..

[2] World Health Organization (2014). World Cancer Report 2014 Chapter 5.1. (www.who.int).

[3] World Health Organization (2014). World Cancer Report 2014 Chapter 1.1. (www.who.int).

[4] Siegel R (2014). CA A Cancer Journal for Clinicians..

[5] Miyamoto S (2015). Metabolites..

[6] Sreekumar A (2009). Nature..

[7] Denkert C (2008). Molecular cancer..

[8] Nishiumi S (2010). Metabolomics..

[9] Kind T (2007). Analytical biochemistry..

[10] Wu H (2009). Analytica Chimica Acta..

[11] Blanchet L, Smolinska A, (2016). Statistical Analysis in Proteomics..

[12] Engle S (2017). BMC bioinformatics..

[13] Xia J (2015). Nucleic acids research..

[14] Kruskal WH, Wallis WA, (1952). Journal of the American Statistical Association..

